# Iodide capped PbS/CdS core-shell quantum dots for efficient long-wavelength near-infrared light-emitting diodes

**DOI:** 10.1038/s41598-017-15244-5

**Published:** 2017-11-07

**Authors:** Xuyong Yang, Fuqiang Ren, Yue Wang, Tao Ding, Handong Sun, Dongling Ma, Xia Wei Sun

**Affiliations:** 1 Key Laboratory of Advanced Display and System Applications of Education of Ministry, Shanghai University, 149 Yanchang Road, Shanghai, 200072 P. R. China; 2 Institut National de la Recherche Scientifique (INRS), Universitédu Québec, 1650 Boulevard Lionel-Boulet, Varennes, Québec, J3X1S2 Canada; 3 Division of Physics and Applied Physics, School of Physical and Mathematical Sciences, Nanyang Technological University, Nanyang Avenue, Singapore, 637371 Singapore; 4 School of Electrical and Electronic Engineering, Nanyang Technological University, Nanyang Avenue, Singapore, 639798 Singapore; 5 Centre for Disruptive Photonic Technologies (CDPT), School of Physical and Mathematical Sciences, Nanyang Technological University, Singapore, 637371 Singapore; 6 Department of Electrical and Electronic Engineering, College of Engineering, Southern University of Science and Technology, 1088 Xue-Yuan Road, Shenzhen, Guangdong, 518055 P. R. China

## Abstract

PbS based quantum dots (QDs) have been studied in great detail for potential applications in electroluminescent devices operating at wavelengths important for telecommunications (1.3–1.6 μm). Despite the recent advances in field of quantum dot light-emitting diode (QLED), further improvements in near-infrared (NIR) emitting device performance are still necessary for the widespread use and commercialization of NIR emitting QLED technology. Here, we report a high-performance 1.51-μm emitting QLED with inverted organic–inorganic hybrid device architecture and PbS/CdS core-shell structured quantum dots as emitter. The resultant QLEDs show a record device performance for the QLEDs in 1.5 μm emission window, with a maximum radiance of 6.04 Wsr^−1^ m^−2^ and peak external quantum efficiency (EQE) of 4.12%, respectively.

## Introduction

Quantum dots (QDs) based light-emitting diodes (QLEDs) have attracted increasing interest due to their narrow emission linewidth, tunable emission spectral window as well as facile solution-processibility^1–4^. QLED has undergone rapid developments both in quantum dot synthesis and device structure optimization since its invention in 1994^5^. Nowadays, QLEDs are emerging as an undeniable competitor to organic light emitting diodes (OLEDs) for lighting and display applications. Despite the huge progress of QLEDs emitting at visible emission range, the development of near-infrared (NIR) emitting QLEDs used in telecommunications is still very slow. Infrared light with a wavelength around 1.3 μm or 1.5 μm, the best emission wavelength choices for standard silica optical fibres because of the low loss transmission response of crystalline silicon at this wavelength range, is an indispensable element of any modern telecommunication network. The extension of OLEDs into NIR spectral range is difficult because organic molecules usually emit light at wavelengths (λ) shorter than 1 μm^6–10^. In contrast, the emission spectrum of quantum dots can be easily adjusted from the visible to NIR range only by simply changing composition and size of quantum dots, which can perfectly matches well with the “transparency window” of silica optical fibre.

Narrow band-gap lead sulfide (PbS) based nanocrystals have been widely studied in the past few years for applications in NIR emitting LEDs operating at wavelengths vital for telecommunications^11–14^. For example, Supran *et al*. reported short-wavelength NIR QLEDs with λ > 1 μm using core-shell structured PbS/CdS QDs^15^. However, previous efforts at developing NIR emitting QLEDs have demonstrated only limited success due to the low emission efficiency and insulating surface ligands of QDs as well as the poor carrier mobility of organic charge transport materials. Recently, Sargent *et al*.^16^ made a breakthrough in the NIR emitting LEDs using PbS QDs as emissive layer. They improved the radiative exciton recombination within the QD layer by embedding QDs in a high-mobility hybrid perovskite matrix to prevent transport-assisted trapping losses. The resultant NIR emitting QLEDs exhibit a two-fold increase in external quantum efficiency (EQE) compared with previous devices. Nevertheless, the highest reported electroluminescence (EL) performance to date has fallen short of those possible with mature technologies for QLEDs emitting at visible emission wavelengths. Meanwhile, the quantum yields of PbS based QDs with short-wavelength emission are generally much lower than those of the QDs with long-wavelength emission because of the emission quenching caused by localized trap states with the particle size increase, which causes the poor performance for NIR QLEDs with long-wavelength emission.

The high performance of QLEDs can be attributed to the concomitant excitation of the luminescent QD film via Förster energy transfer and direct charge injection. The efficiency of moving charges to QDs is strongly dependent on the QD environment. The surface of QDs prepared by traditional colloidal techniques is capped with an insulating layer of organic ligands with long hydrocarbon tails. Here, we report a best-performance 1.51 μm-emitting QLED by employing iodide passivated PbS/CdS core-shell nanocrystals as emissive layer. The charge injection into QD emissive layer is dramatically improved with the use of iodide passivated QDs. Such NIR emitting QLEDs demonstrate the maximum radiance and efficiency of 6.04 Wsr^−1^ m^−2^ and 4.12%, respectively. These results demonstrate a new route towards high-performance NIR emitting QLEDs for telecommunication applications.

## Results

**Figure 1:**
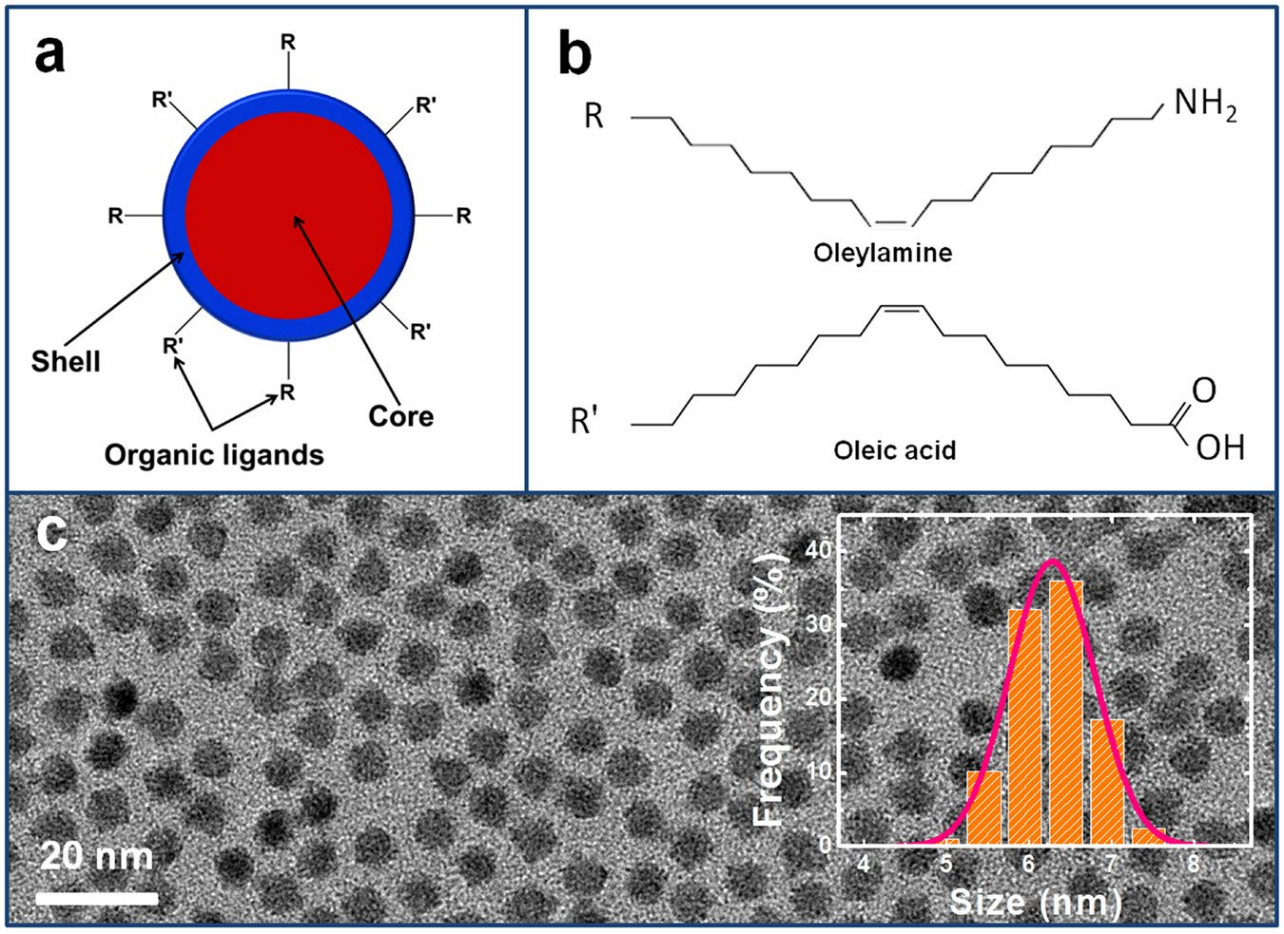
(**a**) Structural description, (**b**) ligand molecular structures, and (**c**) TEM image of the PbS/CdS core-shell QDs.

To achieve high-performance QLEDs, it is necessary to start with a quantum dot emitter with a high quantum yield for electroluminescence where the excited state is generated by electron-hole recombination. In this work, the PbS/CdS core-shell QDs with a ~1481 nm peak emission wavelength we used were prepared by a microwave-assisted cation exchange approach (Supporting Information Fig. S1). The quantum yield of the QDs is ~52%, the highest efficiency reported for PbS-based QDs in this emission range^17^. The surface of QDs is capped with an insulating layer of the mixed oleyamine (OLA) and oleic acid (OA) organic ligands with long hydrocarbon tails which lead to enhanced emission efficiency owing to the effective passivation of surface defects for PbS/CdS QDs (Fig. 1a,b). Figure 1c exhibits the low-magnification transmission electron microscopy (TEM) image of the resultant QDs with a uniform size distribution of an average particle size of ~10 nm.

**Figure 2:**
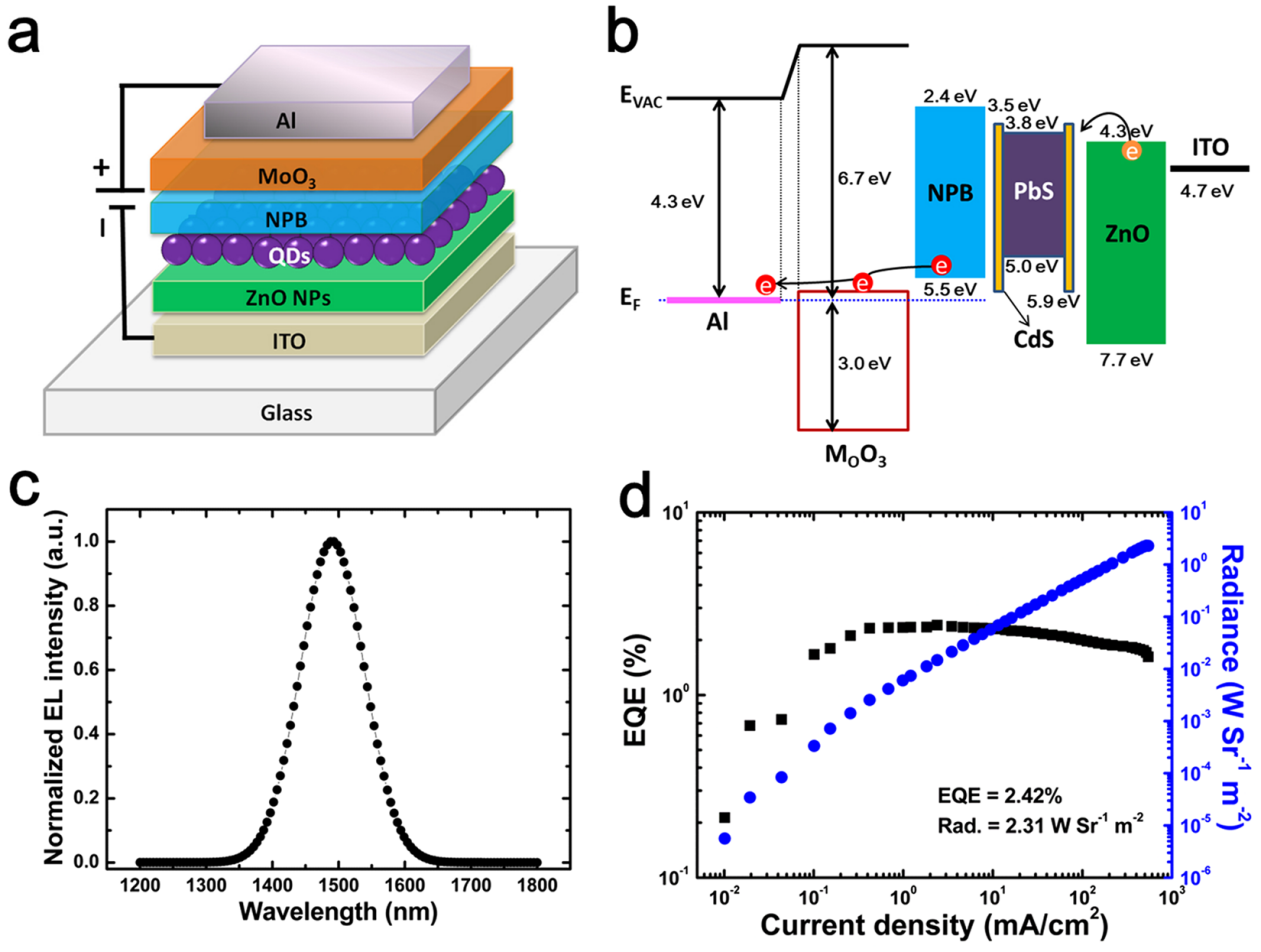
(**a**) Schematic of the device configuration. The theoretical values for the valence and conduction bands for QD layer were obtained from ref.^27^. (**b**) EL spectrum and (**c**) current density-voltage (*J-V*) characteristics of device. Note that the energy levels shown in this figure are absolute values and all the energy values quoted relative to the vacuum level are negative. (**d**) Dependence of radiance and EQE on current density for the NIR QLED with maximum radiance.

We further explored the potential application of these PbS/CdS QDs as emissive layer in NIR QLEDs. The resultant NIR electroluminescent devices comprise a multilayer thin film architecture of ITO/ZnO nanoparticles (NPs)/QDs/4, 4′-N, N’-dicarbazole-biphenyl (NPB)/MoO_3_/Al, as shown in Fig. 2a, where MoO_3_ was used as the hole injection layer (HIL), NPB as the HTL, PbS/CdS core-shell structured QDs as the emissive layer (EML), ZnO NP film spin-casted from ZnO NPs in ethanol solution as the ETL and Al as the anode. The inverted QLED architectures have been demonstrated that they are very efficient for obtaining high QLED device performance and the highest brightness for visible QLEDs was reported by Chang *et al*.^18^ using a similar device architecture with that we used. The schematic energy level diagram of the device depicted in Fig. 2b shows that electrons are injected from the ITO to the QD layer via conduction band of ZnO nanoparticle layer. However, hole injection from Al anode to the organic semiconductor (NPB) results from carrier breakthrough the MoO_3_/NPB junction (the electrons are extracted from the highest occupied molecular orbital (HOMO) level of NPB through the conduction band of MoO_3_). Figure 2c presents the EL spectrum of our optimized NIR emitting QLED. The pure QD emission at a peak wavelength of ~1492 nm illustrates the highly efficient recombination of electrons and holes in the QD emissive layer. The red-shifted peak wavelength (~11 nm) from the QD solution’s photoluminescence (PL) is attributed to the Föster resonant energy transfer from small QDs to the large ones in closely packed QD solids^19^. The radiance and EQE curves as a function of current density for the device are depicted in Fig. 2d. The resultant NIR QLED exhibits an excellent device performance, with a maximum radiance and peak EQE of 2.31 Wsr^−1^ m^−2^ and 2.42%, respectively, which are the highest reported values for the QLEDs in the 1.5 μm emission window.

**Figure 3:**
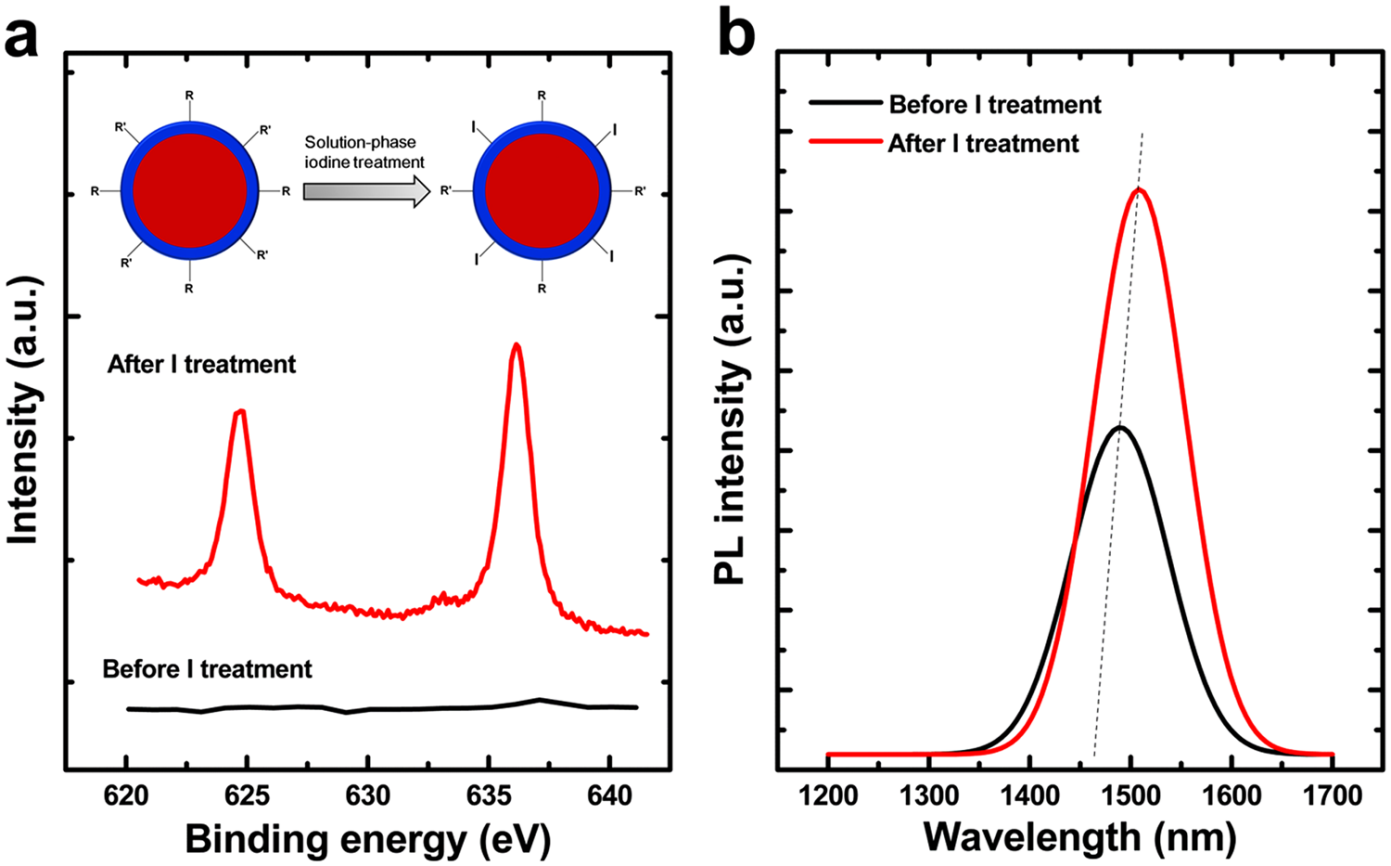
(**a**) Binding energy of I_3d_ in I-capped PbS/CdS QD films. Inset: the ligand exchange procedure to produce solution-phase iodine treated quantum dots. (**b**) The absolute luminescence quantum yield of solution-phase iodine treated QD films *vs*. the untreated QD films.

For inverted QLEDs, the direct charge injection into QD emissive layer is dominant in driving QD EL process compared with Förster energy transfer^20^, and thus this requires facile transport of carriers to QDs. The efficiency of moving charges to QDs is strongly dependent on the QD environment. The surface of QDs prepared by traditional colloidal techniques is generally capped with an insulating layer of organic ligands with long hydrocarbon tails, resulting in the part of inefficient charge injection. It has been previously demonstrated that the inorganic ligands are often more efficient than their organic counterparts^21–23^. In this work, the I ions are connected to QDs for enhancing the surface mobility of QDs by a ligand exchange method, as depicted in the inset of Fig. 3. X-ray photoelectron spectroscopy (XPS) confirmed that the introduction of I ions into the ligands of QDs. As shown by Fig. 3a, strong binding energy peaks of I_3d_ at 624.75 eV and 636.18 eV appeared following the solution-phase halide treatment. We used PL emission spectra (Fig. 3b) to further study the impact of the solution-phase iodine treatment on the physical properties of the QDs. It can be seen that the emission intensity of the I connected QD film is 1.56-fold higher than that of untreated QD film, suggesting that the iodine treatment also plays an important role in passivating the surface defects of PbS based QDs, which is agreement well with the previous findings^24^. In addition, it can be noted that the PL emission peak of QDs with I ligands is red-shifted slightly compared with the untreated QDs due to the strengthening dot-to-dot interaction resulting from the shorter length of I ions than OA/OLA ligands.

**Figure 4:**
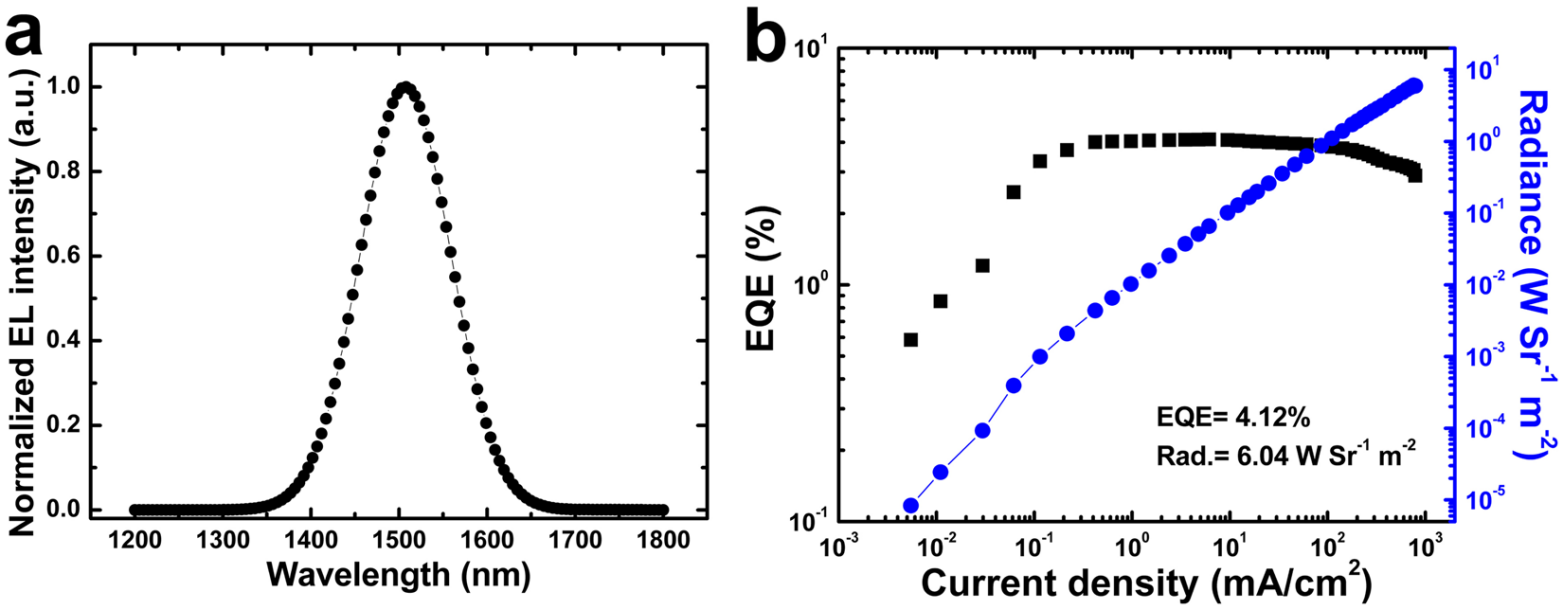
(**a**) EL spectrum and (**b**) dependence of radiance and EQE on current density for the NIR QLED with I treated QDs.

The EL spectrum of the QLED using I passivated QDs with a peak emission of 1510 nm is correspondingly red-shifted ~ 18 nm compared with the above reference device (Fig. 4a). The electrical characterization results reveal that the presence of I ligands in QDs has an obvious effect on the charge transport process that the current injection for the device with I treated QDs is more efficient than that of the reference device (Supporting Information Fig. S2), indicating that the introduction of I ligands facilitates the charge injection into QDs. The significant improvement for the surface properties of QDs contributes to the high performance of our devices. As shown in Fig. 4b, the QLED with iodide passivated PbS/CdS QDs obtained a maximum radiance of 6.04 Wsr^−1^ m^−2^ at the current density of 6.1 mA/cm^2^ and a peak EQE of 4.12% at the current density of 759 mA/cm^2^, the record radiance and efficiency values for NIR QLEDs in the 1.5 μm emission window. In addition, the resulting NIR QLEDs fabricated by the efficient design of inverted structure have excellent device reproducibility (Supporting Information Fig. S3). Nevertheless, it still can be expected that the further optimization such as improving the quantum yields of PbS based QD films would realize better device performances.

## Discussion

In conclusion, we have demonstrated the best-performance NIR-emitting QLEDs with a 1.51 μm wavelength emission. The superior performance in radiance and efficiency was achieved by the use of high-efficiency PbS/CdS core-shell QDs, the surface treatment of QDs with high-mobility inorganic I ions and the efficient design of inverted device architecture. Our results illustrate a further step towards the practical application of NIR emitting QLEDs in optoelectronic devices, especially for telecommunication.

## Methods

### Preparation of iodine treated PbS/CdS QDs and ZnO nanoparticles

Untreated PbS/CdS QDs with core-shell structure were prepared according to a previously reported microwave-assisted cation exchange procedure^17^. For solution-phase iodine treatment, untreated PbS/CdS QDs (30 mg mL^−1^ QDs in toluene (5 mL) were added to tetrabutylammonium iodide (TBAI) solution (0.6 mL), and then the mixed solution was fully stirred for 15–30 minutes. For precipitating the QDs, ethanol was added to centrifuge and separate the QDs solid from the solution. The as-prepared PbS/CdS QDs can be finally dispersed in octane (5 mg mL^−1^). The exchanging process for iodine and organic ligands was carried out in nitrogen glovebox. The TBAI solution^24^ and ZnO NPs^25^ were obtained following previous literature reports.

### Fabrication of NIR emitting QLED devices

The patterned ITO-glass substrates were cleaned by sonication in detergent, de-ionized (DI) water, acetone, and isopropyl alcohol for 15 min. in sequence. Then ZnO NP layer (40 nm) was spin-coated on the cleaned substrates from the ZnO ethanol solution (25 mg/mL) at 1000 rpm for 60 s and baked at 150 °C for 30 min in a nitrogen filled glove box. Next, the 5 mg/mL of PbS/CdS QD toluene solution was spin-coated on the ZnO NP layer at a rate of 2000 rpm for 60 s to form a QD emissive layer (20 nm), and baked at 90 °C for 30 min. Subsequently, the 4, 4′-N, N’-dicarbazole-biphenyl (NPB, 60 nm), MoO_3_ (10 nm) and Al (150 nm) layers were sequentially deposited on the QD layer in a vacuum thermal evaporator.

### Instrumentation

The device electroluminescencec (EL) spectra were collected by a Peltier cooled InGaAs photodiode equipped with a standard lock-in amplifier technique. The overall device performance was measured by using a technique described by Forrest *et al*.^26^ in which a photodiode was covered on the device active pixel. The EL emission of QLEDs measured as the photodiode current and the current of device were simultaneously obtained. The device radiance and external quantum efficiency (EQE) were calculated by these two quantities together with the device EL spectra. Fluorescence spectra of PbS/CdS QDs were recorded with a Fluorolog®-3 system (Horiba JobinYvon) using a photomultiplier tube (PMT) detector and the excitation wavelengths were set at 670 nm. The absolute PL quantum yield (QY) of QDs was measured using an integrating optical sphere. X-ray photoelectron spectroscopy (XPS) data were recorded with a Phiobos 100 spectrometer using Mg X-ray radiation source (SPECS, Germany) at 12.53 kV for the high resolution scans. Transmission electron microscopy (TEM) was characterized by a transmission electron microscope (JEOL, 2100 F) operating at 200 kV.

### Data availability statement

All data generated or analysed during this study are included in this published article (and its Supplementary Information files).

## Electronic supplementary material

Supplementary Information
