# Supplementary material for: Iodide capped PbS/CdS core-shell quantum dots for efficient long-wavelength near-infrared light-emitting diodes
Source: Sci Rep. 2017 Nov 7;7:14741. doi: 10.1038/s41598-017-15244-5 (PMC5677035; doi:10.1038/s41598-017-15244-5)
Supplement: Supplementary file 1 — Supplementary Information [file 41598_2017_15244_MOESM1_ESM.pdf]

Supplementary Information

Iodide capped PbS/CdS core-shell quantum dots for efficient long-wavelength near-infrared light-emitting diodes

Xuyong Yang[1], *Fuqiang Ren*[2], *Yue Wang*[3], *Tao Ding*[4], *Handong Sun*[3,5], *Dongling Ma*[2,*] *and Xiao Wei Sun*[6,*]

[1] Key Laboratory of Advanced Display and System Applications of Education of Ministry, Shanghai University, 149 Yanchang Road, Shanghai 200072, P. R. China

[2] Institut National de la Recherche Scientifique (INRS), Université du Québec, 1650 Boulevard Lionel-Boulet, Varennes, Québec J3X 1S2, Canada

[3] Division of Physics and Applied Physics, School of Physical and Mathematical Sciences, Nanyang Technological University, Nanyang Avenue, Singapore 639798, Singapore

[4] School of Electrical and Electronic Engineering, Nanyang Technological University, Nanyang Avenue, Singapore 639798, Singapore

[5] Centre for Disruptive Photonic Technologies (CDPT), School of Physical and Mathematical Sciences, Nanyang Technological University, Singapore 637371, Singapore

[6] Department of Electrical and Electronic Engineering, Southern University of Science and Technology, 1088 Xue-Yuan Road, Shenzhen, Guangdong 518055, China

[*] To whom correspondence should be addressed. Email: ma@emt.inrs.ca and EXWSun@sustc.edu.sg

SI-I:

Synthesis of PbS/CdS core-shell structured QDs. For a typical QD preparation, CdO (0.9 g), OA (12 mL) and ODE (30 mL) in a three flask were heated to 200–250 °C using an oil bath until the solution became colorless. Next, the solution was cooled to 100 °C and degassed under vacuum for 30 min. The temperature was further decreased to room temperature and 12 mL of PbS QD dispersion was added by a syringe (the PbS QDs were synthesized by using OLA as capping ligand). Then 5 mL of this mixture solution was introduced into a 10 mL reaction tube or a 50 mL flask, and then heated by a microwave reaction or an oil bath to 100 °C for 10 min. To purify the PbS/CdS QDs, the QDs were extracted by the addition of ethanol, followed by centrifugation. The precipitate was subsequently redispersed in toluene and again precipitated with ethanol.

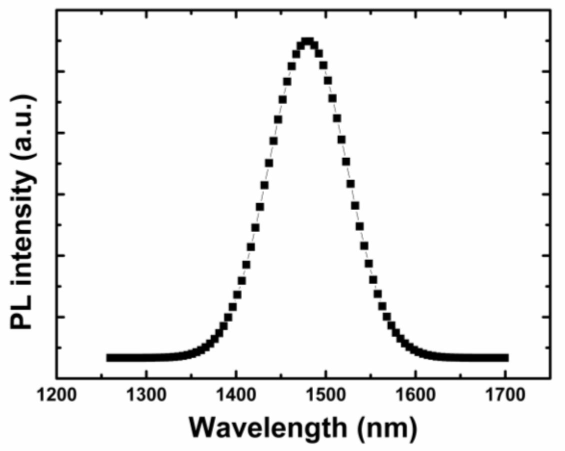

Fig. S1. QD PL emission. PL spectrum of the resultant PbS/CdS core-shell structured QDs.

The sizes of the PbS QDs and the PbS cores in core-shell QDs were calculated based on their bandgap energy estimated from the first exciton absorption peak.[1-3]

(1) Moreels, I.; Lambert, K.; Smeets, D.; De Muynck, D.; Nollet, T.; Martins, J. C.; Vanhaecke, F.; Vantomme, A.; Delerue, C.; Allan, G. *ACS Nano* **2009**, *3*, 3023.

(2) Moreels, I.; Justo, Y.; De Geyter, B.; Haustraete, K.; Martins, J. C.; Hens, Z. *ACS Nano* **2011**, *5*, 2004.

(3) Cademartiri, L.; Montanari, E.; Calestani, G.; Migliori, A.; Guagliardi, A.; Ozin, G. A. *J. Am. Chem. Soc.* **2006**, *128*, 10337.

SI-2:

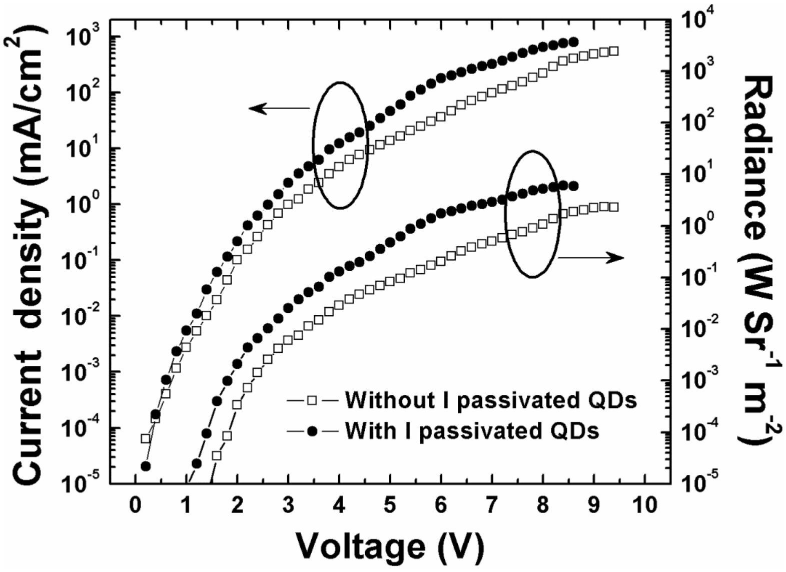

Fig. S2. Electric characteristics. Current density-voltage-radiance (*J-V-R*) characteristics for QLEDs with I treated QDs and without I treated QDs.

It can be noted that Figure S2 does not show the typical diode characteristics for the resulting NIR QLED devices, which is possibly caused by the existing of a particular functional layer such as small organic-molecule ligands/surface defects of ZnO nanoparticles those are sensitive to electric field. Under an applied bias, the electrically unstable materials in device influence carrier injection and thus result in the atypical diode characteristics.

SI-3:

Device reproducibility test was performed. As shown by the histograms for 21 devices (Fig. S3), the highest EQE value for the resulting QLEDs reaches 4.12% and an average EQE is 3.85% with a standard deviation of 0.411%.

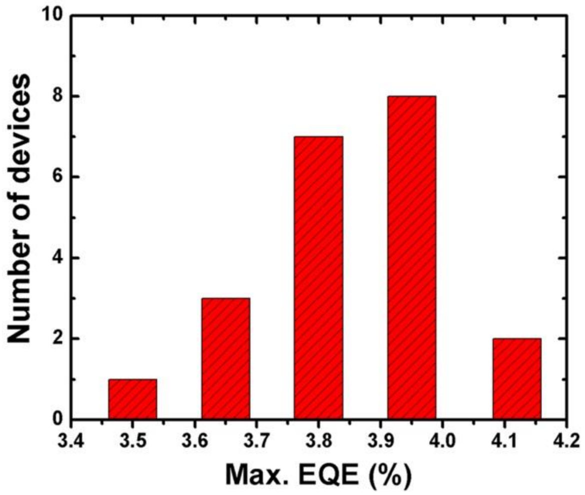

Fig. S3. Histograms of peak EQEs of the resulting NIR QLEDs with the same fabrication condition.
